# Predictors of Low Cardiac Output Syndrome in Infants After Open-Heart Surgery

**DOI:** 10.3389/fped.2022.829731

**Published:** 2022-03-10

**Authors:** Liang Zou, Di Yu, Ruonan Wang, Yueshuang Cun, Yaping Li, Qingfeng Wang, Yaqin Shu, Xuming Mo

**Affiliations:** Department of Cardiothoracic Surgery, Children's Hospital of Nanjing Medical University, Nanjing, China

**Keywords:** low cardiac output syndrome, cardiopulmonary bypass, aortic cross-clamping time, body weight, thyroid hormones

## Abstract

**Objective:**

To evaluate the predictors of low cardiac output syndrome (LCOS) in infants with congenital heart disease (CHD) after cardiopulmonary bypass (CPB).

**Study design:**

A total of 217 infants were enrolled and classified according to whether they developed LCOS after cardiac surgery. Each infant's preoperative and intraoperative clinical variables were collected.

**Results:**

The incidence of LCOS was 28.11% in our study. The univariate analysis showed that the LCOS group was younger than the non-LCOS group (25.69 ± 25.01 days vs. 44.45 ± 26.97 days, *P* < 0.001), with a higher proportion of neonates (60.7 vs. 27.6%, *P* < 0.001) and a higher proportion of patients with a RACHS-1 score ≥4 (50.8 vs. 17.9%, *P* < 0.001). A lower weight (3.70 ± 0.74 vs. 4.23 ± 1.10 kg, *P* = 0.001), longer ACC time (61.96 ± 21.44 min vs. 41.06 ± 18.37 min, *P* < 0.001) and longer CPB time (131.54 ± 67.21 min vs. 95.78 ± 62.67 min, *P* < 0.001) were found in the LCOS group. The levels of free triiodothyronine (FT3) (4.55 ± 1.29 pmol/L vs. 5.18 ± 1.42 pmol/L, *P* = 0.003) and total triiodothyronine (TT3) (1.80 ± 0.56 nmol/L vs. 1.98 ± 0.54 nmol/L, *P* = 0.026) were also lower in the LCOS group. The multivariate binary logistic regression analysis and receiver operating characteristic (ROC) indicated that the ACC time, FT3 level and body weight were independent predictors of LCOS.

**Conclusions:**

In our patient population, we first propose that preoperative FT3 can predict the occurrence of postoperative LCOS. ACC time, FT3 level and body weight are independent predictors of LCOS and maybe helpful in reducing the incidence of postoperative LCOS in the future.

## Introduction

Low cardiac output syndrome (LCOS) is a common, well-recognized collection of physiological and hemodynamic disturbances indicating the inability of the heart to deliver sufficient oxygen to tissue and end organs to meet metabolic demand. LCOS was first reported by Parr et al. (1) and typically occurs in 25% to 65% of children with congenital heart defects (CHDs) 6–8 h after cardiopulmonary bypass (CPB) (2). LCOS contributes to a longer duration of postoperative mechanical ventilation (MV), prolonged intensive care unit (ICU) length of stay, and increased mortality (3). Therefore, preventing this predictable syndrome may have important implications for improving clinical outcomes.

The key to preventing LCOS during the postoperative period is early recognition and timely intervention. Du et al. found that the CPB temperature, myocardial protection with histidine-tryptophan-ketoglutarate, and postoperative application of a residual shunt were independent risk predictors of LCOS in children older than 3 months (4). Additionally, a small number of cytokines, such as the preoperative neutrophil-lymphocyte ratio, postoperative mid-regional pro-adrenomedullin (MR-proADM) and postoperative cardiac troponin I (cTn-I), have been reported as risk factors in children who presented with LCOS after cardiac surgery (5, 6). However, the risk predictors of LCOS in infants following CHD surgery under CPB are still uncertain. Therefore, we conducted a retrospective study to identify the predictors of postoperative LCOS in infants under 3 months undergoing CHD surgery with CPB.

## Materials and Methods

### Study Population

This study protocol was approved by the Institutional Ethical Committees of the Children's Hospital of Nanjing Medical University. In this retrospective study, 217 infants with CHD following cardiac surgery under CPB were enrolled from June 2017 to March 2021. Patients were excluded on the basis of the following criteria: (1) cardiac surgery without CPB; (2) an age over 3 months at the time of surgery; (3) trisomy 21/18 syndrome; (4) death during surgery or within the first 6 h after surgery; (5) previous cardiac surgery; (6) presence of significant extra cardiac anomalies, congenital renal anomalies, airway anomalies, or gastrointestinal anomalies; and (7) preoperative myocardial dysfunction, acidosis and shock.

### Clinical Data

We collected clinical variables that may affect postoperative LCOS, preoperative variables including sex, age at the time of surgery, premature, weight at the time of surgery, Risk Adjustment in Congenital Heart Surgery-1 (RACHS-1) score (7), type of CHD, pulmonary arterial pressure (PAH), preoperative levels of thyroid hormones [total T3 (TT3, normal rage: 1.29–3.11 nmol/L), free T3 (FT3, normal rage: 2.8–7.1 pmol/L), total T4 (TT4, normal rage: 66–187.4 nmol/L), free T4 (FT4, normal rage: 12.1–22 pmol/L), and thyroid-stimulating hormone (TSH, normal rage: 0.2–5 μIU/ml)], preoperative levels of albumin, blood urea nitrogen (BUN), serum creatinine (Scr), and alanine transaminase (ALT), intraoperative variables including CPB time, aortic cross-clamping (ACC) time, and postoperative variables including 24-h postoperative vasoactive-inotropic score (VIS), and ICU mortality. The patients were classified according to whether they developed LCOS after corrective surgery (LCOS group and non-LCOS group). LCOS was diagnosed when two or more of the following criteria were met (6, 8, 9): tachycardia (heart rate >90th percentile of normal), hypotension (systolic blood pressure < *p*5 for age and sex), oliguria (urine output <1 mL/kg per hour lasting more than 2 h), peripheral skin temperature to core body temperature difference of >7°C, a continuously increased lactic acid level (change rate >0.75 mmol/L per hour), central venous oxygen saturation (ScvO2) <0%, left ventricular ejection fraction as determined by Doppler echocardiography <40%, and a first 24 h peak vasoactive-inotropic score (VIS) ≥20.

### Statistical Analysis

The statistical analysis was performed using SPSS version 20.0 software (Chicago, IL, USA). Continuous variables are expressed as the mean ± standard deviation, while categorical variables are summarized as frequencies and percentages. Comparisons between two groups were performed using an unpaired Student's *t*-test for the continuous variables and a χ2 or Fisher's exact test for the categorical variables. In the above univariate analysis, each parameter with *P* ≤ 0.1 was included in the multiple logistic regression analysis to identify the independent predictors of postoperative LCOS. Receiver operating characteristic (ROC) curves were used to examine the variables, and the area under the curve (AUC) was calculated from the ROC curve. The results are presented with the sensitivity and specificity based on the AUC. Statistical significance was defined as *P* < 0.05.

## Results

### Patient Characteristics

We retrospectively reviewed 217 infants under 3 months old [mean age, 39.16 ± 27.70 days; 139 boys (64.1%); 80 neonates (36.9%)]. Seventeen patients were premature and their age at time of surgery was adjusted according to their gestational age, and there was no difference in the proportion of preterm infants between the two groups. The mean weight of the enrolled infants was 4.08 ± 1.04 kg. Among these patients, 59 infants (27.1%) had a RACHS-1 score ≥4. In total, 151 infants (69.6%) presented with preoperative PAH. The mean CPB and ACC times were 105.82 ± 65.03 min and 46.93 ± 21.39 min, respectively. The preoperative levels of thyroid hormones, including FT3, FT4, TT3, TT4, and TSH, were 5.00 ± 1.42 pmol/L, 20.78 ± 5.84 pmol/L, 1.93 ± 0.55 nmol/L, 120.07 ± 32.97 nmol/L and 5.21 ± 5.41 μIU/ml, respectively. The demographic and physiological characteristics of the patients are shown in Table 1.

**Table 1:** Characteristics of enrolled patients with congenital heart disease.

**Characteristic**	**All patients (*n* = 217)**
**Age (days)**	39.16 ± 27.70
Neonate	80 (36.9%)
Infant	137 (63.1%)
**Premature**	17 (7.8%)
**Female**	78 (35.9%)
**Weight (kg)**	4.08 ± 1.04
**RACHS-1**	
Score-1	5 (2.3%)
Score-2	66 (30.4%)
Score-3	87 (40.1%)
Score-4	55 (25.3%)
Score-5	4 (1.8%)
Score-6	0 (0%)
**Type of Surgery**	
ASD	3 (1.4%)
VSD	21 (9.7%)
VSD + ASD	80 (36.9%)
COA/IAA	29 (13.4%)
TAPVC	34 (15.7%)
TGA	27 (10.4%)
PA-VSD	5 (2.3%)
DORV	4 (1.8%)
Other	14 (6.5%)
**Operative factors**	
CPB time (min)	105.82 ± 65.03
ACC time (min)	46.93 ± 21.39
**PAH**	151 (69.6%)
**ICU mortality**	18 (8.3%)
**Pre-operative thyroid function**	
FT3 (pmol/L)	5.00 ± 1.42
FT4 (pmol/L)	20.78 ± 5.84
TT3 (nmol/L)	1.93 ± 0.55
TT4 (nmol/L)	120.07 ± 32.97
TSH (μIU/ml)	5.21 ± 5.41
**Other preoperative parameters**	
ALT (U/L)	16.21 ± 11.51
ALB (g/L)	39.93 ± 6.79
BUN (mmol/L)	9.42 ± 3.19
Scr (μmol/L)	42.21 ± 19.24

*RACHS-1, risk adjustment in congenital heart surgery-1; ASD, atrial septal defect; VSD, ventricular septal defect; COA, coarctation of aorta; IAA, interrupter aortic arch; TAPVC, total anomalous pulmonary venous connection; TGA, transposition of the great arteries; PA, pulmonary atresia; DORV, double outlet right ventricular; CPB, cardiopulmonary bypass; ACC, aortic cross-clamping; PAH, pulmonary hypertension; FT3, free triiodothyronine; FT4, free thyroxin; TT3, total triiodothyronine; TT4, total thyroxin; TSH, thyroid stimulating hormone; ALT, alanine transaminase; ALB, albumin; BUN, blood urea nitrogen; Scr, serum creatinine*.

### Comparison of the LCOS and Non-LCOS Groups

Sixty-one infants presented with LCOS (28.11%) according to the LCOS criteria mentioned above. The LCOS group was younger than the non-LCOS group (25.69 ± 25.01 days vs. 44.45 ± 26.97 days, *P* < 0.001), with a higher proportion of neonates (37/61 vs. 43/156, *P* < 0.001) and a higher proportion of patients with RACHS-1 scores ≥4 (31/61 vs. 28/156, *P* < 0.001). Compared with the non-LCOS group, the LCOS group had a lower weight (3.70 ± 0.74 kg vs. 4.23 ± 1.10 kg, *P* = 0.001), longer ACC time (61.96 ± 21.44 min vs. 41.06 ± 18.37 min, *P* < 0.001) and longer CPB time (131.54 ± 67.21 min vs. 95.78 ± 62.67 min, *P* < 0.001). The levels of free triiodothyronine (FT3) (4.55 ± 1.29 pmol/L vs. 5.18 ± 1.42 pmol/L, *P* = 0.003) and total triiodothyronine (TT3) (1.80 ± 0.56 nmol/L vs. 1.98 ± 0.54 nmol/L, *P* = 0.026) in the LCOS group were lower than those in the non-LCOS group. In addition, the LCOS group had low levels of FT4 and TT4 and a high TSH level, but no significant differences were observed between the groups. The LCOS group exhibited a higher level of Scr (46.70 ± 21.40 μmol/L vs. 40.45 ± 18.09 μmol/L, *P* = 0.031) than the non-LCOS group. No significant differences in the proportion of individuals with PAH or levels of albumin, ALT or BUN were observed between the groups (Table 2).

**Table 2:** Characteristics of patients according to LCOS group and non-LCOS group.

**Characteristic**	**Non-LCOS** **(*N* = 156)**	**LCOS** **(*N* = 61)**	***P*-value**
Age (days)	44.45 ± 26.97	25.69 ± 25.01	**<0.001**
Neonate	43 (27.6%)	37 (60.7%)	**<0.001**
Premature	10 (6.4%)	7 (11.5)	0.212
Female	62 (39.7%)	16 (26.2%)	0.083
Weight (kg)	4.23 ± 1.10	3.70 ± 0.74	**0.001**
RACHS-1≥4	28 (17.9%)	31 (50.8%)	**<0.001**
PAH	111(71.2%)	40(65.6%)	0.512
CPB time (min)	95.78 ± 62.67	131.54 ± 67.21	**<0.001**
ACC time (min)	41.06 ± 18.37	61.96 ± 21.44	**<0.001**
FT3 (pmol/L)	5.18 ± 1.42	4.55 ± 1.29	**0.003**
FT4 (pmol/L)	20.83 ± 5.94	20.66 ± 5.60	0.850
TT3 (nmol/L)	1.98 ± 0.54	1.80 ± 0.56	**0.026**
TT4 (nmol/L)	120.73 ± 32.85	118.40 ± 33.49	0.641
TSH (μIU/ml)	4.86 ± 4.43	6.01 ± 7.31	0.220
ALT (U/L)	15.05 ± 6.31	19.16 ± 19.01	0.103
ALB (g/L)	40.36 ± 6.36	38.85 ± 7.74	0.141
BUN (mmol/L)	9.55 ± 3.06	9.10 ± 3.52	0.351
Scr (μmol/L)	40.45 ± 18.09	46.70 ± 21.40	**0.031**
VIS	12.72 ± 4.05	24.88 ± 7.04	**<0.001**

*RACHS-1, risk adjustment in congenital heart surgery-1; ASD, atrial septal defect; VSD, ventricular septal defect; COA, coarctation of aorta; IAA, interrupter aortic arch; TAPVC, total anomalous pulmonary venous connection; TGA, transposition of the great arteries; PA, pulmonary atresia; DORV, double outlet right ventricular; CPB, cardiopulmonary bypass; ACC, aortic cross-clamping; PAH, pulmonary hypertension; FT3, free triiodothyronine; FT4, free thyroxin; TT3, total triiodothyronine; TT4, total thyroxin; TSH, thyroid stimulating hormone; ALT, alanine transaminase; ALB, albumin; BUN, blood urea nitrogen; Scr, serum creatinine. The bold values indicate that the results have statistical significance*.

### Independent Predictors of LCOS

Each parameter with *P* ≤ 0.1 was included in the multiple logistic regression analysis to identify the independent predictors of postoperative LCOS, and the ACC time, FT3, and weight were found to be independent predictors as shown in Table 3.

**Table 3:** Multivariate logistic regression, odds ratio of variables for predicting LCOS in infants with CHD after CPB.

**Variables**	**B**	**S.E**.	**Wald**	**df**	***P*-value**	**OR**	**95% CI. for OR**	
							**Lower**	**Upper**
Age	−0.009	0.009	1.122	1	0.290	0.991	0.974	1.008
Gender	0.225	0.409	0.301	1	0.583	1.252	0.561	2.793
Weight	−0.498	0.238	4.375	1	**0.036**	0.607	0.381	0.969
RASCH-1≥ 4	−0.519	0.448	1.347	1	0.246	0.595	0.247	1.430
CPB time	0.005	0.004	1.687	1	0.194	1.005	0.998	1.012
ACC time	0.034	0.013	6.767	1	**0.009**	1.034	1.008	1.061
FT3	−0.641	0.267	5.766	1	**0.016**	0.527	0.312	0.889
TT3	0.959	0.661	2.105	1	0.147	2.609	0.714	9.528
Scr	−0.009	0.011	0.775	1	0.379	0.991	0.971	1.011
Constant	0.911	1.286	0.502	1	0.479	2.486		

*RACHS-1, risk adjustment in congenital heart surgery-1; FT3, free triiodothyronine; CPB, cardiopulmonary bypass; ACC, aortic cross-clamping; TT3, total triiodothyronine; Scr, serum creatinine. The bold values indicate that the results have statistical significance*.

### Efficacy of Weight, ACC Time, and FT3 in Predicting LCOS

**Figure 1:**
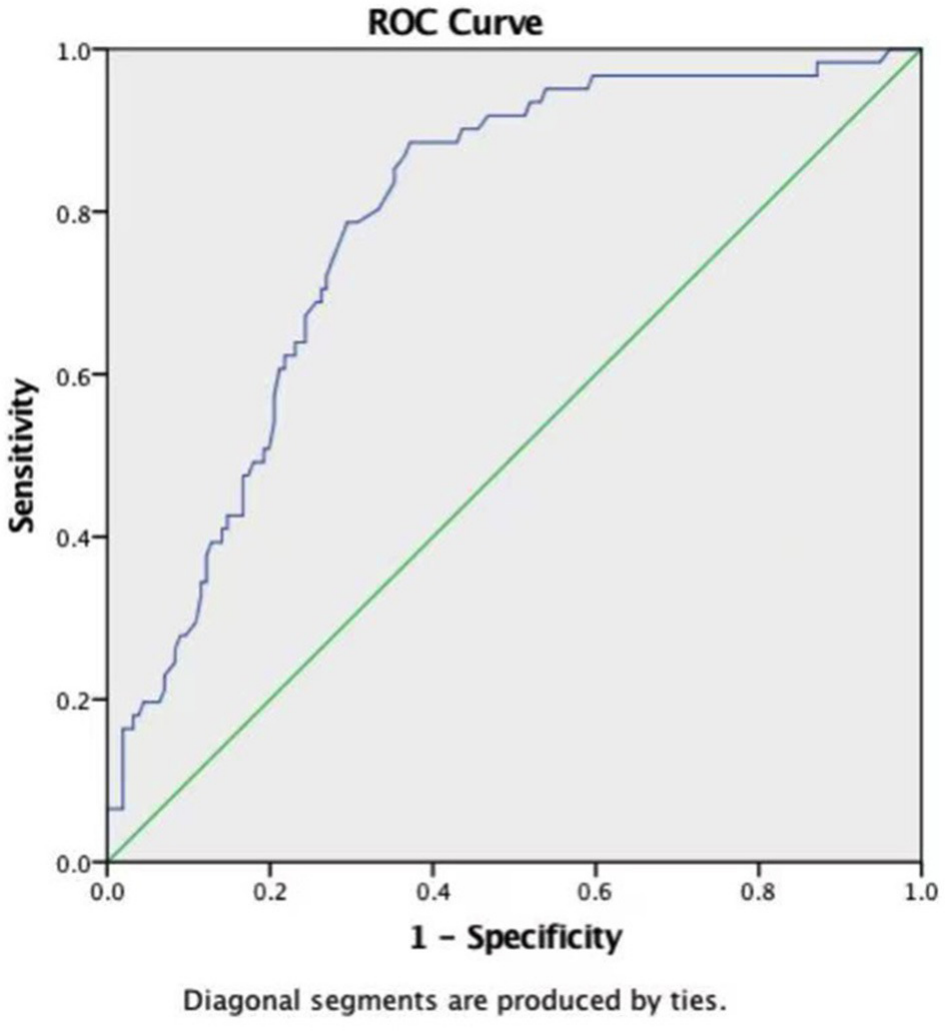
Receiver operating characteristic curves of aortic cross-clamping (ACC time).

**Figure 2:**
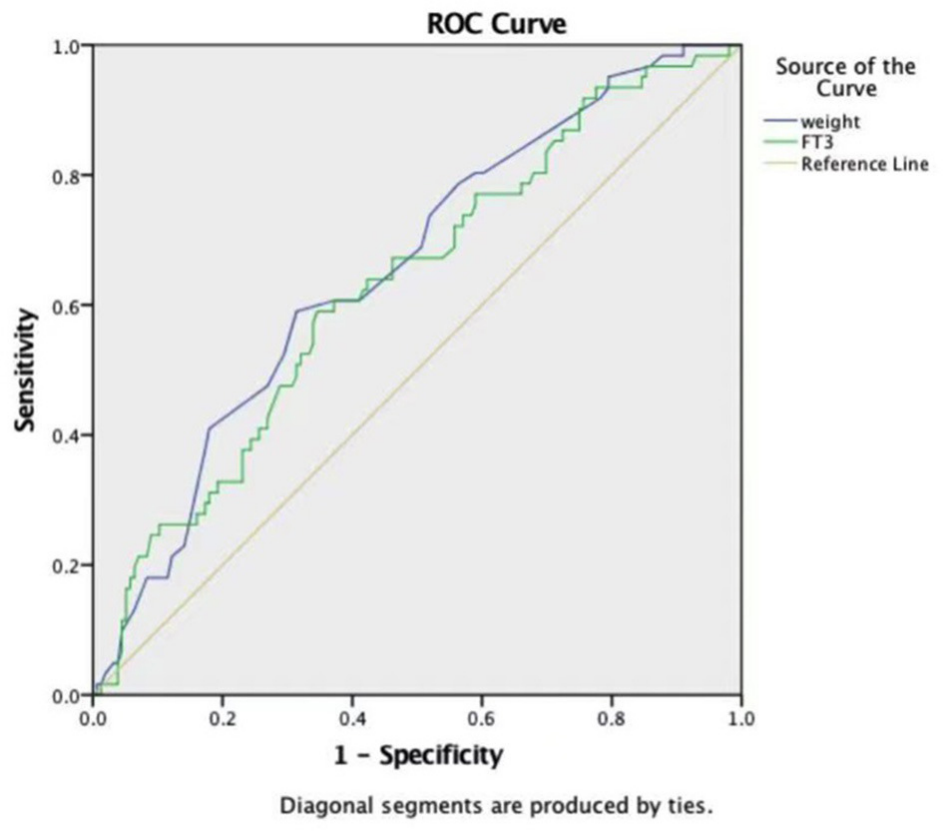
Receiver operating characteristic curves of weight and free triiodothyronine (FT3).

ROC curves were constructed to evaluate the potential ability of weight, the ACC time, and FT3 to predict the development of LCOS (Table 4). The ROC curve analysis of the ACC time showed a good ability to predict LCOS development with a cutoff of 40.65 min (AUC: 0.786 ± 0.033; sensitivity: 88.5%; specificity 62.8%; *p* < 0.001) (Table 4, Figure 1). Although characterized by slightly lower AUCs, the weight and FT3 ROC curves were also moderately predictive of LCOS development (AUCs: 0.656 ± 0.040 and 0.631 ± 0.042; cutoff values: 3.75 kg and 4.695 pmol/L; sensitivity: 59 and 59%; specificity 68.6 and 65.4%, respectively; Table 4, Figure 2).

**Table 4:** Area under the curve of variables and values to predict postoperative LCOS.

**Variables**	**AUC-ROC**	***P*-value**	**Cutoff value**	**Sensitivity (%)**	**Specificity (%)**
Weight (kg)	0.656 ± 0.040	<0.001	≤ 3.75	59	68.6
FT3 (pmol/L)	0.631 ± 0.042	0.003	≤ 4.695	59	65.4
ACC time (min)	0.786 ± 0.033	<0.001	≥40.65	88.5	62.8
Predictor model	0.832 ± 0.030	<0.001			

*AUC-ROC, area under the receiver operating characteristic curve; FT3, free triiodothyronine; CPB, cardiopulmonary bypass; ACC, aortic cross-clamping*.

## Discussion

The incidence of LCOS in our study was 28.11%, which is higher than that in a retrospective cohort study involving children with a median age of 11 months old (9.98%) (4), and similar to another study with a 25.9% incidence of LCOS (10). Age may account for this difference. The proportion of newborns included in this study was larger (36.9%), and previous studies have shown that neonates were more prone to LCOS (11–13). However, in the multiple logistic regression analysis showed that the age was not the risk factor for LCOS. This may be due to the small age difference (range 1 to 90 days) in our study.

We found that the preoperative FT3 level, body weight and ACC time may outperform other clinical parameters in predicting the incidence of postoperative LCOS in infants less than 3 months old undergoing CHD surgery with CPB. To the best of our knowledge, we are the first to propose that the preoperative FT3 level predicts the incidence of postoperative LCOS.

Thyroid hormones play an important role in improving cardiac output by increasing the cardiac index and lowering systemic vascular resistance (14). Thyroid hormone levels are markedly suppressed in children and adults after cardiac surgery associated with ultrafiltration and hemodilution during CPB (15, 16). Plumpton et al. demonstrated that younger children with longer CPB time showed lower FT3 levels (17). Mainwaring et al. found that neonatal TSH, FT3, and thyroglobulin levels decreased by 80% after CPB (18). Additionally, CPB is well-known to cause systemic inflammatory response syndrome, and inflammatory cytokines such as IL-6 have been shown to inhibit peripheral conversion of T4 to T3 (19). Abnormal circulating thyroid hormone levels are found during systemic disease, especially after cardiac surgery with CBP in the absence of primary thyroid disease. This is collectively known as Euthyroid sick syndrome (ESS). ESS is considered a non-thyroidal illness syndrome (20, 21) and may cause LCOS (22). Marwali et al. (23) performed a randomized placebo-controlled double-blind clinical trial and found that postoperative triiodothyronine supplementation reduced the LCOS incidence and altered the distribution of LCOS during the postoperative period. Portman et al. (24) also found that thyroid supplementation significantly lowered inotropic use and improved cardiac function on echocardiography in patients younger than 5 months. However, in this study, we focused on the relationship between the preoperative thyroid hormone level and LCOS incidence and found that an FT3 level ≤ 4.695 pmol/L was an independent predictor of postoperative LCOS among other thyroid hormones. A prospective, double-blind, randomized placebo-controlled clinical pilot trial conducted by Zhang et al. (25) showed that the level of postoperative FT3 can be accordingly increased by increasing the level of FT3 before CPB. There was a certain correlation between the preoperative FT3 level and postoperative FT3 level. Therefore, the preoperative FT3 levels may affect the occurrence of LCOS. The FT3 levels were reported to be affected by albumin, cortisol, and heparin (26–28). In our study, the patients were not preoperatively administered cortisol or heparin, and no difference in the preoperative albumin levels was observed between the groups. Therefore, the preoperative level of FT3 was considered a relatively reliable independent predictor of postoperative LCOS according to the analysis of the ROC curves and AUC.

We also found that weight was another moderate independent predictor of LCOS in this study. The advantages of delaying operation to allow young infants to gain weight remain controversial. Some researchers propose that delaying surgery in clinically stable infants may result in improved survival (29, 30). In contrast, other researchers reported that the rate of preoperative complications is obviously related to the duration of waiting (31) and suggest that early operation did not compromise survival (32, 33). However, we observed that a weight ≤ 3.75 kg during the operation predicted the incidence of postoperative LCOS in the ROC and AUC analyses. Lu et al. (34) found that a low operation weight was a significant risk factor for mortality in infants with congenital heart defects in a Chinese population. Therefore, we suggest that if there are no other factors indications for emergency surgery, body weight should be increased by strengthening nutrition, which could help reduce the incidence of postoperative LCOS.

Drennan et al. (35) reported that the best predictor of postoperative LCOS was the ACC time, which is consistent with our finding. We also found that an ACC time ≥40.65 min was the most powerful independent predictor of LCOS. Myocardial ischemia-reperfusion induced by cross-clamping of the aorta plays an important role in the development of postoperative LCOS in pediatric patients (22). In our study, the LCOS group had a longer ACC time than the non-LCOS group, indicating that the LCOS group suffered from more severe myocardial ischemia and injury (36). CPB is usually closely related to postoperative LCOS (37), while a longer CPB time contributes to an increased dependence on inotropic support (38). In our study, although the CPB time in the LCOS group was significantly longer than that in the non-LCOS group, the CPB time could not be used as a predictor of postoperative LCOS. We considered that sufficient and effective myocardial protection during CPB was the primary influencing factor (39).

In this study, the potential drawbacks include the retrospective design, the enrollment of patients from a single site, and the application of the clinical diagnostic criteria for LCOS without invasive cardiac output monitoring, which could interfere with our results. In addition, our inclusion criteria were mainly for children with low left cardiac output syndrome without considering the impact of right ventricular insufficiency on left ventricular function. We will further study this issue in our future work.

In conclusion, we found that the preoperative FT3 level, ACC time and body weight were independent predictors of postoperative LCOS through multivariate regression and ROC curve analyses. To the best of our knowledge, this study is the first time to propose that preoperative FT3 can predict the occurrence of postoperative LCOS. These predictors could provide intervention targets for clinicians in the future and could be helpful in reducing the incidence of postoperative LCOS. Further multicenter investigations in neonates and infants should be designed as randomized controlled trials to confirm the role of these three predictors in the occurrence of postoperative LCOS.

## Data Availability Statement

The raw data supporting the conclusions of this article will be made available by the authors, without undue reservation.

## Ethics Statement

The studies involving human participants were reviewed and approved by Institutional Ethical Committees of the Children's Hospital of Nanjing Medical University. Written informed consent to participate in this study was provided by the participants' legal guardian/next of kin.

## Author Contributions

XM and YS designed the study. LZ and DY wrote the manuscript. LZ, DY, and RW collected the data, and YC, YL, and QW analyzed the data. All authors reviewed the manuscript and approved the submitted version.

## Funding

This research was supported by the Science and Technology Development Fund Key Project of Nanjing Medical University (NMUB2019214, to LZ).

## Conflict of Interest

The authors declare that the research was conducted in the absence of any commercial or financial relationships that could be construed as a potential conflict of interest.

## Publisher's Note

All claims expressed in this article are solely those of the authors and do not necessarily represent those of their affiliated organizations, or those of the publisher, the editors and the reviewers. Any product that may be evaluated in this article, or claim that may be made by its manufacturer, is not guaranteed or endorsed by the publisher.
